# {4,4′-Dimeth­oxy-2,2′-[2,2-dimethyl­propane-1,3-diylbis(nitrilo­methyl­idyne)]diphenolato}nickel(II)

**DOI:** 10.1107/S1600536809019965

**Published:** 2009-06-06

**Authors:** Morteza Montazerozohori, Mohammad Hossein Habibi, Reza Mokhtari, Yuki Yamane, Takayoshi Suzuki

**Affiliations:** a Department of Chemistry, Yasouj University, Yasouj 75914-353, Iran; b Catalysis Division, Department of Chemistry, University of Isfahan, Isfahan 81746-73441, Iran; c Department of Chemistry, Faculty of Science, Okayama University, Tsushima-naka 3-1-1, Okayama 700-8530, Japan

## Abstract

In the title complex, [Ni(C_21_H_24_N_2_O_4_)], the Ni^II^ ion has a slightly distorted square-planar geometry, coordinated by the two N and two O atoms of a new tetra­dentate Schiff base ligand. The dihedral angle between the planes of the two NiNC_3_O chelate rings is 14.37 (12)°.

## Related literature

For the structures of free Schiff bases, see: Garnovskii *et al.* (1993 ▶). Nickel(II) complexes with N_2_O_2_ Schiff-base ligands derived from salicylaldehyde have long been used as homogenous catalysts (Gosden *et al.*, 1981 ▶; Healy & Pletcher, 1978 ▶). For related structures, see: Habibi *et al.* (2007*a* ▶,*b* ▶). For Ni—O and Ni—N distances, see: Akhtar (1981 ▶); Shkolnikova *et al.* (1970 ▶).
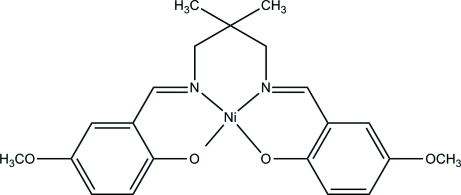


## Experimental

### 

#### Crystal data

[Ni(C_21_H_24_N_2_O_4_)]
*M* *_r_* = 427.13
Orthorhombic, 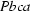

*a* = 15.6110 (7) Å
*b* = 9.1151 (5) Å
*c* = 26.8142 (12) Å
*V* = 3815.5 (3) Å^3^
*Z* = 8
Mo *K*α radiation
μ = 1.05 mm^−1^
*T* = 193 K
0.30 × 0.20 × 0.20 mm

#### Data collection

Rigaku R-AXIS RAPID diffractometer
Absorption correction: multi-scan (*ABSCOR*; Higashi, 1995 ▶) *T*_min_ = 0.744, *T*_max_ = 0.818
35644 measured reflections
4362 independent reflections
3946 reflections with *I* > 2σ(*I*)
*R*_int_ = 0.020

#### Refinement

*R*[*F* ^2^ > 2σ(*F* ^2^)] = 0.026
*wR*(*F* ^2^) = 0.071
*S* = 1.05
4362 reflections
258 parameters
H-atom parameters constrained
Δρ_max_ = 0.43 e Å^−3^
Δρ_min_ = −0.18 e Å^−3^

### 

Data collection: *PROCESS-AUTO* (Rigaku, 1998 ▶); cell refinement: *PROCESS-AUTO*; data reduction: *CrystalStructure* (Rigaku/MSC, 2004 ▶); program(s) used to solve structure: *SIR2004* (Burla *et al.*, 2005 ▶); program(s) used to refine structure: *SHELXL97* (Sheldrick, 2008 ▶); molecular graphics: *ORTEP-3 for Windows* (Farrugia, 1997 ▶); software used to prepare material for publication: *SHELXL97*.

## Supplementary Material

Crystal structure: contains datablocks I, global. DOI: 10.1107/S1600536809019965/bt2968sup1.cif

Structure factors: contains datablocks I. DOI: 10.1107/S1600536809019965/bt2968Isup2.hkl

Additional supplementary materials:  crystallographic information; 3D view; checkCIF report

## Figures and Tables

**Fig. 1.**
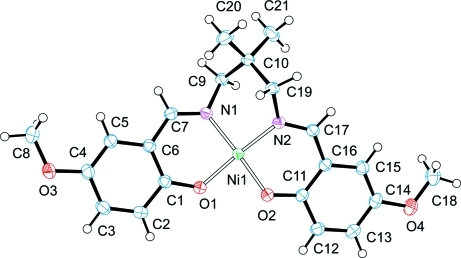
A view of the title compound, showing the atom-numbering scheme. Displacement ellipsoids are drawn at the 30% probability level and H atoms are shown as small spheres of arbitrary radii.

## supplementary crystallographic information

### Comment

Schiff bases and their biologically active complexes have been studied extensively over the past decade. Although numerous transition metal complexes of Schiff bases have been structurally characterized, relatively few free Schiff bases have been similarly characterized (Garnovskii *et al.*, 1993).

Nickel(II) complexes with N_2_O_2_ Schiff-base ligands derived from salicylaldehyde have long been used as homogenous catalysts (Gosden *et al.*, 1981; Healy & Pletcher, 1978).

Recently we reported the structure of a copper(II) and nickel(II) complexes with the *N*,*N*'-bis(6-methoxysalicylidene)-1,3-diaminopropane ligand (Habibi *et al.*, 2007*a*,b). The title compound is isostructural with its Cu^II^ and Ni^II^ analogues.

In the title compound (Figure 1), the Ni—O and Ni—N distances are larger than the comparable mean distances of 1.829 and 1.859 Å (Table 1), respectively, in *N*,*N*'-ethylenebis(salicylideneiminato)nickel(II) (Shkolnikova *et al.*, 1970) and 1.849 (2) and 1.840 (2) Å, respectively, in *N*,*N*'-ethylenebis[(2-hydroxy-1-naphthyl)methaniminato]nickel(II) (Akhtar, 1981).

### Experimental

A mixture of 6-methoxysalicylaldehyde (2.0 mmol, 304 mg) and 2,2-dimethylpropane-1,3-diamine (1.0 mmol, 102 mg) was dissolved in methanol (10 ml) with stirring for 15 min at room temperature, to give a clear yellow solution. A methanol solution (10 ml) of Ni(OAc)_2_.4H_2_O (1.0 mmol, 249 mg) was then added. The mixture was refluxed for a further 45 min and then filtered. After keeping the filtrate in air for 5 d, dark green block-shaped crystals were formed at the bottom of the vessel on slow evaporation of the solvent, in about 85% yield.

### Refinement

All H atoms were placed in geometrically idealized positions and allowed to ride on their parent atoms, with C—H distances in the range 0.93–0.97 Å and with *U*_iso_(H) = 1.2 or 1.5 times *U*_eq_(C).

### Crystal data

**Table:**

[Ni(C_21_H_24_N_2_O_4_)]	*D*_x_ = 1.487 Mg m^−^^3^
*M**_r_* = 427.13	Mo *K*α radiation, λ = 0.71075 Å
Orthorhombic, *P**b**c**a*	Cell parameters from 27241 reflections
*a* = 15.6110 (7) Å	θ = 3.0–27.5°
*b* = 9.1151 (5) Å	µ = 1.05 mm^−^^1^
*c* = 26.8142 (12) Å	*T* = 193 K
*V* = 3815.5 (3) Å^3^	Block, dark-green
*Z* = 8	0.30 × 0.20 × 0.20 mm
*F*(000) = 1792	

### Data collection

**Table:**

Rigaku R-AXIS RAPID diffractometer	4362 independent reflections
Radiation source: fine-focus sealed tube	3946 reflections with *I* > 2σ(*I*)
graphite	*R*_int_ = 0.020
Detector resolution: 10.00 pixels mm^-1^	θ_max_ = 27.5°, θ_min_ = 3.0°
ω scans	*h* = −20→20
Absorption correction: multi-scan (*ABSCOR*; Higashi, 1995)	*k* = −11→11
*T*_min_ = 0.744, *T*_max_ = 0.818	*l* = −34→32
35644 measured reflections	

### Refinement

**Table:**

Refinement on *F*^2^	Primary atom site location: structure-invariant direct methods
Least-squares matrix: full	Secondary atom site location: difference Fourier map
*R*[*F*^2^ > 2σ(*F*^2^)] = 0.026	Hydrogen site location: inferred from neighbouring sites
*wR*(*F*^2^) = 0.071	H-atom parameters constrained
*S* = 1.05	*w* = 1/[σ^2^(*F*_o_^2^) + (0.0413*P*)^2^ + 1.3733*P*] where *P* = (*F*_o_^2^ + 2*F*_c_^2^)/3
4362 reflections	(Δ/σ)_max_ = 0.001
258 parameters	Δρ_max_ = 0.43 e Å^−^^3^
0 restraints	Δρ_min_ = −0.18 e Å^−^^3^

### Special details

**Table:**

Geometry. All e.s.d.'s (except the e.s.d. in the dihedral angle between two l.s. planes) are estimated using the full covariance matrix. The cell e.s.d.'s are taken into account individually in the estimation of e.s.d.'s in distances, angles and torsion angles; correlations between e.s.d.'s in cell parameters are only used when they are defined by crystal symmetry. An approximate (isotropic) treatment of cell e.s.d.'s is used for estimating e.s.d.'s involving l.s. planes.
Refinement. Refinement of *F*^2^ against ALL reflections. The weighted *R*-factor *wR* and goodness of fit *S* are based on *F*^2^, conventional *R*-factors *R* are based on *F*, with *F* set to zero for negative *F*^2^. The threshold expression of *F*^2^ > σ(*F*^2^) is used only for calculating *R*-factors(gt) *etc*. and is not relevant to the choice of reflections for refinement. *R*-factors based on *F*^2^ are statistically about twice as large as those based on *F*, and *R*- factors based on ALL data will be even larger.

### Fractional atomic coordinates and isotropic or equivalent isotropic displacement parameters (Å^2^)

**Table:**

	*x*	*y*	*z*	*U*_iso_*/*U*_eq_	
Ni1	0.951753 (10)	0.180679 (18)	0.014687 (6)	0.02025 (7)	
O1	0.85263 (6)	0.10007 (11)	0.04139 (3)	0.0281 (2)	
O2	0.91005 (6)	0.10714 (11)	−0.04479 (3)	0.0271 (2)	
O3	0.75989 (7)	0.00704 (12)	0.23785 (4)	0.0353 (2)	
O4	1.01038 (8)	0.20756 (14)	−0.23918 (4)	0.0398 (3)	
N1	0.97892 (7)	0.27883 (12)	0.07416 (4)	0.0215 (2)	
N2	1.05801 (7)	0.23220 (13)	−0.01347 (4)	0.0209 (2)	
C1	0.83516 (8)	0.08183 (15)	0.08876 (5)	0.0234 (3)	
C2	0.76375 (9)	−0.00620 (16)	0.10235 (5)	0.0287 (3)	
H2	0.7304	−0.0512	0.0770	0.034*	
C3	0.74193 (9)	−0.02755 (16)	0.15133 (5)	0.0304 (3)	
H3	0.6940	−0.0874	0.1592	0.036*	
C4	0.78912 (9)	0.03727 (15)	0.19010 (5)	0.0273 (3)	
C5	0.85834 (8)	0.12301 (15)	0.17879 (5)	0.0252 (3)	
H5	0.8908	0.1669	0.2048	0.030*	
C6	0.88167 (8)	0.14645 (14)	0.12825 (5)	0.0227 (3)	
C7	0.94728 (8)	0.25124 (15)	0.11773 (5)	0.0229 (3)	
H7	0.9696	0.3053	0.1451	0.028*	
C8	0.79861 (12)	0.0897 (2)	0.27678 (6)	0.0436 (4)	
H8A	0.7692	0.0693	0.3083	0.052*	
H8B	0.7942	0.1946	0.2691	0.052*	
H8C	0.8591	0.0623	0.2797	0.052*	
C9	1.03568 (8)	0.40646 (14)	0.06956 (5)	0.0231 (3)	
H9A	1.0336	0.4635	0.1009	0.028*	
H9B	1.0144	0.4704	0.0424	0.028*	
C10	1.12843 (8)	0.36428 (15)	0.05867 (5)	0.0238 (3)	
C11	0.93847 (8)	0.13593 (15)	−0.08973 (5)	0.0232 (3)	
C12	0.88895 (9)	0.08927 (15)	−0.13120 (5)	0.0272 (3)	
H12	0.8368	0.0382	−0.1255	0.033*	
C13	0.91455 (9)	0.11618 (16)	−0.17925 (5)	0.0290 (3)	
H13	0.8794	0.0852	−0.2062	0.035*	
C14	0.99180 (10)	0.18878 (15)	−0.18915 (5)	0.0289 (3)	
C15	1.04265 (9)	0.23295 (16)	−0.15007 (5)	0.0272 (3)	
H15	1.0956	0.2807	−0.1565	0.033*	
C16	1.01630 (9)	0.20742 (14)	−0.10023 (5)	0.0228 (3)	
C17	1.07511 (8)	0.23987 (14)	−0.06053 (5)	0.0226 (2)	
H17	1.1313	0.2695	−0.0697	0.027*	
C18	1.08696 (13)	0.2857 (2)	−0.24969 (6)	0.0474 (4)	
H18A	1.0940	0.2950	−0.2859	0.057*	
H18B	1.1360	0.2325	−0.2357	0.057*	
H18C	1.0838	0.3836	−0.2347	0.057*	
C19	1.13172 (8)	0.23648 (15)	0.02101 (5)	0.0230 (3)	
H19A	1.1851	0.2446	0.0012	0.028*	
H19B	1.1341	0.1428	0.0396	0.028*	
C20	1.17187 (10)	0.31209 (18)	0.10659 (6)	0.0349 (3)	
H20A	1.2295	0.2761	0.0988	0.042*	
H20B	1.1381	0.2328	0.1215	0.042*	
H20C	1.1759	0.3940	0.1302	0.042*	
C21	1.17386 (10)	0.49902 (17)	0.03763 (6)	0.0364 (3)	
H21A	1.2348	0.4769	0.0329	0.044*	
H21B	1.1678	0.5811	0.0610	0.044*	
H21C	1.1482	0.5256	0.0055	0.044*	

### Atomic displacement parameters (Å^2^)

**Table:**

	*U*^11^	*U*^22^	*U*^33^	*U*^12^	*U*^13^	*U*^23^
Ni1	0.01948 (10)	0.02097 (10)	0.02030 (10)	−0.00186 (6)	0.00049 (6)	−0.00188 (6)
O1	0.0253 (5)	0.0358 (5)	0.0231 (4)	−0.0077 (4)	0.0010 (4)	−0.0026 (4)
O2	0.0279 (5)	0.0305 (5)	0.0229 (4)	−0.0065 (4)	0.0015 (4)	−0.0025 (4)
O3	0.0381 (6)	0.0421 (6)	0.0258 (5)	−0.0117 (5)	0.0024 (5)	0.0073 (4)
O4	0.0459 (7)	0.0524 (7)	0.0211 (5)	−0.0077 (5)	−0.0003 (5)	0.0034 (4)
N1	0.0194 (5)	0.0200 (5)	0.0250 (5)	−0.0007 (4)	0.0011 (4)	−0.0019 (4)
N2	0.0207 (5)	0.0189 (5)	0.0230 (5)	0.0012 (4)	−0.0007 (4)	−0.0017 (4)
C1	0.0212 (6)	0.0239 (6)	0.0253 (6)	0.0006 (5)	0.0014 (5)	−0.0005 (5)
C2	0.0258 (7)	0.0303 (7)	0.0302 (7)	−0.0059 (5)	−0.0019 (6)	−0.0016 (5)
C3	0.0267 (7)	0.0312 (7)	0.0332 (7)	−0.0076 (5)	0.0014 (6)	0.0037 (6)
C4	0.0290 (7)	0.0281 (7)	0.0248 (6)	−0.0009 (5)	0.0019 (5)	0.0047 (5)
C5	0.0245 (6)	0.0275 (7)	0.0236 (6)	−0.0004 (5)	−0.0014 (5)	0.0009 (5)
C6	0.0209 (6)	0.0223 (6)	0.0248 (6)	0.0005 (5)	0.0010 (5)	−0.0006 (5)
C7	0.0217 (6)	0.0235 (7)	0.0236 (6)	0.0005 (5)	−0.0012 (5)	−0.0031 (5)
C8	0.0474 (9)	0.0601 (11)	0.0234 (7)	−0.0133 (8)	−0.0003 (7)	0.0057 (7)
C9	0.0246 (6)	0.0191 (6)	0.0255 (6)	−0.0018 (5)	0.0024 (5)	−0.0028 (5)
C10	0.0213 (6)	0.0239 (6)	0.0262 (6)	−0.0031 (5)	0.0005 (5)	−0.0032 (5)
C11	0.0256 (6)	0.0198 (6)	0.0242 (6)	0.0024 (5)	0.0003 (5)	−0.0024 (5)
C12	0.0257 (6)	0.0267 (7)	0.0292 (7)	−0.0003 (5)	−0.0016 (5)	−0.0041 (5)
C13	0.0321 (7)	0.0295 (7)	0.0254 (6)	0.0030 (6)	−0.0053 (6)	−0.0042 (5)
C14	0.0356 (8)	0.0293 (7)	0.0219 (6)	0.0026 (6)	0.0003 (6)	0.0011 (5)
C15	0.0291 (7)	0.0263 (7)	0.0262 (7)	−0.0006 (5)	0.0019 (5)	0.0007 (5)
C16	0.0256 (6)	0.0200 (6)	0.0230 (6)	0.0016 (5)	−0.0004 (5)	−0.0018 (5)
C17	0.0226 (6)	0.0196 (6)	0.0257 (6)	−0.0004 (5)	0.0019 (5)	−0.0018 (5)
C18	0.0581 (11)	0.0578 (11)	0.0264 (7)	−0.0113 (9)	0.0073 (8)	0.0074 (7)
C19	0.0190 (6)	0.0244 (6)	0.0256 (6)	0.0011 (5)	−0.0022 (5)	−0.0024 (5)
C20	0.0314 (7)	0.0428 (9)	0.0304 (7)	0.0021 (6)	−0.0072 (6)	−0.0077 (6)
C21	0.0331 (8)	0.0281 (7)	0.0480 (9)	−0.0089 (6)	0.0117 (7)	−0.0044 (6)

### Geometric parameters (Å, °)

**Table:**

Ni1—O2	1.8483 (9)	C9—C10	1.5264 (17)
Ni1—O1	1.8566 (9)	C9—H9A	0.9900
Ni1—N1	1.8770 (11)	C9—H9B	0.9900
Ni1—N2	1.8821 (11)	C10—C21	1.5263 (19)
O1—C1	1.3098 (16)	C10—C20	1.529 (2)
O2—C11	1.3107 (16)	C10—C19	1.5425 (18)
O3—C4	1.3869 (16)	C11—C16	1.4072 (18)
O3—C8	1.4222 (19)	C11—C12	1.4196 (19)
O4—C14	1.3833 (17)	C12—C13	1.3710 (19)
O4—C18	1.420 (2)	C12—H12	0.9500
N1—C7	1.2932 (17)	C13—C14	1.401 (2)
N1—C9	1.4676 (16)	C13—H13	0.9500
N2—C17	1.2917 (17)	C14—C15	1.375 (2)
N2—C19	1.4765 (16)	C15—C16	1.4174 (19)
C1—C6	1.4124 (18)	C15—H15	0.9500
C1—C2	1.4211 (18)	C16—C17	1.4368 (18)
C2—C3	1.371 (2)	C17—H17	0.9500
C2—H2	0.9500	C18—H18A	0.9800
C3—C4	1.404 (2)	C18—H18B	0.9800
C3—H3	0.9500	C18—H18C	0.9800
C4—C5	1.3678 (19)	C19—H19A	0.9900
C5—C6	1.4195 (18)	C19—H19B	0.9900
C5—H5	0.9500	C20—H20A	0.9800
C6—C7	1.4286 (18)	C20—H20B	0.9800
C7—H7	0.9500	C20—H20C	0.9800
C8—H8A	0.9800	C21—H21A	0.9800
C8—H8B	0.9800	C21—H21B	0.9800
C8—H8C	0.9800	C21—H21C	0.9800
			
O2—Ni1—O1	84.02 (4)	C21—C10—C20	110.79 (12)
O2—Ni1—N1	170.21 (5)	C9—C10—C20	109.78 (11)
O1—Ni1—N1	92.82 (4)	C21—C10—C19	110.51 (11)
O2—Ni1—N2	93.14 (4)	C9—C10—C19	110.31 (10)
O1—Ni1—N2	171.13 (5)	C20—C10—C19	107.48 (11)
N1—Ni1—N2	91.30 (5)	O2—C11—C16	124.67 (12)
C1—O1—Ni1	126.72 (8)	O2—C11—C12	118.43 (12)
C11—O2—Ni1	126.98 (9)	C16—C11—C12	116.88 (12)
C4—O3—C8	115.62 (11)	C13—C12—C11	121.59 (13)
C14—O4—C18	115.53 (12)	C13—C12—H12	119.2
C7—N1—C9	117.43 (11)	C11—C12—H12	119.2
C7—N1—Ni1	126.06 (9)	C12—C13—C14	120.89 (13)
C9—N1—Ni1	116.29 (8)	C12—C13—H13	119.6
C17—N2—C19	116.69 (11)	C14—C13—H13	119.6
C17—N2—Ni1	125.98 (9)	C15—C14—O4	125.58 (14)
C19—N2—Ni1	116.25 (8)	C15—C14—C13	119.40 (13)
O1—C1—C6	124.53 (12)	O4—C14—C13	115.01 (13)
O1—C1—C2	118.93 (12)	C14—C15—C16	120.19 (13)
C6—C1—C2	116.53 (12)	C14—C15—H15	119.9
C3—C2—C1	121.38 (13)	C16—C15—H15	119.9
C3—C2—H2	119.3	C11—C16—C15	121.02 (12)
C1—C2—H2	119.3	C11—C16—C17	119.92 (12)
C2—C3—C4	121.28 (13)	C15—C16—C17	118.64 (12)
C2—C3—H3	119.4	N2—C17—C16	125.50 (12)
C4—C3—H3	119.4	N2—C17—H17	117.2
C5—C4—O3	125.32 (13)	C16—C17—H17	117.2
C5—C4—C3	119.38 (13)	O4—C18—H18A	109.5
O3—C4—C3	115.30 (12)	O4—C18—H18B	109.5
C4—C5—C6	120.03 (12)	H18A—C18—H18B	109.5
C4—C5—H5	120.0	O4—C18—H18C	109.5
C6—C5—H5	120.0	H18A—C18—H18C	109.5
C1—C6—C5	121.40 (12)	H18B—C18—H18C	109.5
C1—C6—C7	119.97 (12)	N2—C19—C10	113.82 (11)
C5—C6—C7	118.23 (12)	N2—C19—H19A	108.8
N1—C7—C6	125.60 (12)	C10—C19—H19A	108.8
N1—C7—H7	117.2	N2—C19—H19B	108.8
C6—C7—H7	117.2	C10—C19—H19B	108.8
O3—C8—H8A	109.5	H19A—C19—H19B	107.7
O3—C8—H8B	109.5	C10—C20—H20A	109.5
H8A—C8—H8B	109.5	C10—C20—H20B	109.5
O3—C8—H8C	109.5	H20A—C20—H20B	109.5
H8A—C8—H8C	109.5	C10—C20—H20C	109.5
H8B—C8—H8C	109.5	H20A—C20—H20C	109.5
N1—C9—C10	112.90 (10)	H20B—C20—H20C	109.5
N1—C9—H9A	109.0	C10—C21—H21A	109.5
C10—C9—H9A	109.0	C10—C21—H21B	109.5
N1—C9—H9B	109.0	H21A—C21—H21B	109.5
C10—C9—H9B	109.0	C10—C21—H21C	109.5
H9A—C9—H9B	107.8	H21A—C21—H21C	109.5
C21—C10—C9	107.99 (11)	H21B—C21—H21C	109.5
			
O2—Ni1—O1—C1	167.96 (12)	C9—N1—C7—C6	170.27 (12)
N1—Ni1—O1—C1	−21.33 (12)	Ni1—N1—C7—C6	−4.06 (19)
N2—Ni1—O1—C1	96.3 (3)	C1—C6—C7—N1	−11.4 (2)
O1—Ni1—O2—C11	168.52 (11)	C5—C6—C7—N1	175.86 (13)
N1—Ni1—O2—C11	97.0 (3)	C7—N1—C9—C10	112.05 (13)
N2—Ni1—O2—C11	−19.91 (11)	Ni1—N1—C9—C10	−73.06 (12)
O2—Ni1—N1—C7	87.7 (3)	N1—C9—C10—C21	161.18 (11)
O1—Ni1—N1—C7	16.86 (11)	N1—C9—C10—C20	−77.94 (14)
N2—Ni1—N1—C7	−155.28 (11)	N1—C9—C10—C19	40.32 (15)
O2—Ni1—N1—C9	−86.7 (3)	Ni1—O2—C11—C16	12.44 (19)
O1—Ni1—N1—C9	−157.53 (9)	Ni1—O2—C11—C12	−169.17 (9)
N2—Ni1—N1—C9	30.33 (9)	O2—C11—C12—C13	179.71 (13)
O2—Ni1—N2—C17	14.38 (12)	C16—C11—C12—C13	−1.8 (2)
O1—Ni1—N2—C17	85.4 (3)	C11—C12—C13—C14	1.2 (2)
N1—Ni1—N2—C17	−156.90 (12)	C18—O4—C14—C15	−3.2 (2)
O2—Ni1—N2—C19	−153.30 (9)	C18—O4—C14—C13	177.78 (14)
O1—Ni1—N2—C19	−82.3 (3)	C12—C13—C14—C15	0.3 (2)
N1—Ni1—N2—C19	35.43 (9)	C12—C13—C14—O4	179.37 (13)
Ni1—O1—C1—C6	13.03 (19)	O4—C14—C15—C16	179.91 (13)
Ni1—O1—C1—C2	−168.29 (10)	C13—C14—C15—C16	−1.1 (2)
O1—C1—C2—C3	−179.21 (13)	O2—C11—C16—C15	179.35 (13)
C6—C1—C2—C3	−0.4 (2)	C12—C11—C16—C15	0.93 (19)
C1—C2—C3—C4	0.3 (2)	O2—C11—C16—C17	6.8 (2)
C8—O3—C4—C5	9.4 (2)	C12—C11—C16—C17	−171.57 (12)
C8—O3—C4—C3	−170.61 (14)	C14—C15—C16—C11	0.5 (2)
C2—C3—C4—C5	−0.2 (2)	C14—C15—C16—C17	173.07 (13)
C2—C3—C4—O3	179.77 (14)	C19—N2—C17—C16	166.28 (12)
O3—C4—C5—C6	−179.65 (13)	Ni1—N2—C17—C16	−1.35 (19)
C3—C4—C5—C6	0.3 (2)	C11—C16—C17—N2	−12.5 (2)
O1—C1—C6—C5	179.26 (13)	C15—C16—C17—N2	174.77 (13)
C2—C1—C6—C5	0.56 (19)	C17—N2—C19—C10	121.25 (13)
O1—C1—C6—C7	6.7 (2)	Ni1—N2—C19—C10	−69.89 (13)
C2—C1—C6—C7	−171.96 (12)	C21—C10—C19—N2	−90.70 (14)
C4—C5—C6—C1	−0.5 (2)	C9—C10—C19—N2	28.64 (15)
C4—C5—C6—C7	172.12 (12)	C20—C10—C19—N2	148.30 (11)
